# The Clinical Role of Electrocardiographic Morphology of Premature Ventricular Contractions for Prognostic Outcomes in Children

**DOI:** 10.3390/medicina62061165

**Published:** 2026-06-16

**Authors:** Rita Kunigeliene, Germanas Marinskis, Vytautas Usonis, Odeta Kinciniene

**Affiliations:** 1 Clinic of Children’s Diseases, Institute of Clinical Medicine, Faculty of Medicine, Vilnius University, Universiteto St. 3, 01513 Vilnius, Lithuania; 2 Clinic of Cardiac and Vascular Diseases, Institute of Clinical Medicine, Faculty of Medicine, Vilnius University, Universiteto St. 3, 01513 Vilnius, Lithuania

**Keywords:** premature ventricular contractions, electrocardiography, morphology, children

## Abstract

*Background and Objectives:* Premature ventricular contractions are among the most common arrhythmias encountered in clinical practice. However, this disorder can be associated with arrhythmia-induced cardiomyopathy or be the first sign of primary myocardial diseases. Certain morphologies of premature ventricular contractions are associated with a higher risk for sudden arrhythmia and cardiac dysfunction in the adult population. There is data on the clinical value and significance of the contraction morphology in adults, but there is a lack of such data for children. *Materials and Methods:* This observational prospective study of pediatric outpatients with premature ventricular contractions was conducted at Vilnius University Hospital Santaros Clinics. Inclusion criteria comprised children aged 3–17 years with more than 5% premature ventricular contractions over 24 h. Exclusion criteria included previously diagnosed congenital heart defects and cardiomyopathies, channelopathies, or the presence of any acute condition. The electrocardiographic morphology and measurements were assessed, analyzed, and described in this study. *Results:* The electrocardiograms of 80 patients were analyzed according to the ECG-estimated morphology of the arrhythmia complex, arrhythmic QRS complex duration, ratio with the normal QRS complex, and maximum deflection index in V5–V6 derivations. Cardiac MRI abnormalities (8 of 30 MRI studies) was reliably associated with a PVC duration of >150 ms and the maximal amount of extrasystoles per 24 h, with a median amount of 29.6%. A long postcoupling interval (>0.9 s) was associated with PVC progression. *Conclusions:* In this exploratory pediatric cohort, wider PVC QRS duration and higher maximal PVC burden were associated with ventricular MRI abnormalities, while longer postcoupling interval was associated with PVC progression.

## 1. Introduction

Premature ventricular contractions (PVCs) are common findings in clinical practice, occurring in 1–20% of the general population [1]. PVCs can be found in structurally abnormal and normal hearts, with mostly idiopathic causes. In pediatrics, idiopathic PVCs are considered benign conditions with a good prognosis [2,3,4].

PVCs can originate from many locations, including the outflow tracts, ventricular free wall, ventricular septum, aortic cusp, tricuspid/mitral annuli, pulmonary artery, or papillary muscles, and all display unique QRS morphologies on 12-lead electrocardiograms (12-lead ECGs) [5]. However, recent data suggests that a high burden of PVCs with specific characteristics may significantly increase a patient’s risk of developing PVC-induced cardiomyopathy [5]. Frequent, or specifically localized, ventricular extrasystoles can result in prolonged left ventricular (LV) asynchrony and, even in a structurally healthy heart, may cause myocardial dysfunction, left ventricular dilatation, and heart failure—known as PVC-induced cardiomyopathy [6,7]. Certain PVCs characteristics—including specific origins (such as non-outflow tract or papillary muscle sites), high PVC burden, complexity (e.g., couplets, triplets, or non-sustained ventricular tachycardia), multifocal origin, and increased frequency during physical exertion—may raise suspicion for underlying structural heart disease [8].

PVCs may also be a sign of underlying cardiac pathology [8]. In individuals with heart diseases, multifocal and LV PVCs are associated with a poor prognosis [9]. The PVC QRS duration is also used to identify high-risk PVC patients [9]. PVCs with wider QRS complexes are associated with the development of cardiomyopathy in adults [9]. There is debate regarding the duration of PVCs’ QRS complexes being associated with the development of cardiomyopathy in adults, with values ranging from 140 to 157 ms in various studies [9]. There is data on the clinical value and significance of PVC morphology in adults, but such data is lacking for children.

The aim of this study was to determine the most accurate electrocardiographic characteristics of PVCs as a predictor of clinical deterioration in pediatric patients.

## 2. Materials and Methods

### 2.1. Participants and Procedures

This prospective observational study involving children with structurally normal hearts and premature ventricular contractions was approved by the Regional Ethics Committee (No. 2021/10-1383-859(1)). We received signed informed consent forms from the parents or guardians of each participant, and/or from the children if they were aged ≥12 years.

Inclusion criteria comprised children aged 3–17 years with a PVCs burden of at least 5% per 24 h and/or complex PVCs (couplets, triplets, or non-sustained ventricular tachycardia) and/or multiform PVCs documented on a 12-lead ECG. Exclusion criteria included hemodynamically significant heart disease, cardiomyopathies, channelopathies, any acute medical condition, or refusal to participate in the study. This study was performed in the Vilnius University Hospital Santaros Clinics from 1 January 2022 to 30 May 2025.

The characteristics of the patients, including their gender, age, and symptoms (e.g., palpitations, dizziness, fatigue, chest pain, or syncope), were recorded.

All children also underwent several (every 6 months) 24 h ECGs by Holter where the total counts of PVCs and complex (e.g., pairs, triplets, non-sustained ventricular tachycardias), multiform PVCs were assessed. We included the results of the initial test, the test with the highest number of PVCs (maximal PVCs count), and the last 24 h ECG performed during the study.

Transthoracic echocardiography was performed using a Philips EPIQ 7 ultrasound system in accordance with the 2014 ACC/AAP/AHA/ASE/HRS/SCAI/SCCT/SCMR/SOPE recommendations for initial transthoracic echocardiographic evaluation in outpatient pediatric cardiology [10]. The echocardiographic assessment included evaluation for structural, septal, valvular, and myocardial abnormalities. Minor valvular regurgitation and small patent foramen ovale shunts were considered hemodynamically insignificant. Left ventricular ejection fraction (LVEF) was measured using the biplane Simpson method from apical four- and two-chamber views. Left ventricular systolic dysfunction was defined as LVEF below 55%.

Cardiac magnetic resonance imaging (MRI) was performed according to indications in the international cardiovascular MRI guidelines [11]. Cardiac MRI measurements were indexed to body surface area and interpreted according to age- and sex-specific z-score nomograms [12]. Ventricular dilatation was defined as a ventricular diastolic diameter greater than 2 z-scores based on nomograms [12]. Abnormal ventricular function was defined as LVEF < 55% [11,12,13,14,15]. Myocardial fibrosis was detected on MRI as late myocardial accumulation of contrast material [11,16].

Cardiogenetic testing was performed only in patients with suspected ion channel disease and/or cardiomyopathy according to the Heart Rhythm Society and European Heart Rhythm Association expert consensus (2011) [17].

### 2.2. 12-Lead ECG

We obtained 12-lead ECGs using the BTL FLEXI ECG, device, (BTL Industries, Prague, Czech Republic) and BTL CardioPointsoftware CardioPoint 3 (BTL Industries, Prague, Czech Republic). All 12-lead ECGs were recorded at a speed of 25 mm/s and an amplitude of 10 mm/mV. Standard leads I, II, and III; amplified unipolar limb leads aVR, aVL, and aVF; and unipolar thoracic leads V1, V2, V3, V4, V5, and V6 were recorded.

We assessed the following 12-lead ECG parameters:

(1) ECG-estimated anatomical localization of the PVCs was determined according to algorithms.

We divided the possible PVC localization according to the algorithms into three variants (Table 1).

**Variant N1** (Table 1):

- Left bundle branch block (LBBB)-morphology PVCs;
- Right bundle branch block (RBBB)-morphology PVCs.

**Variant N2** (Table 1):

- Outflow tract PVCs;
- Non-outflow tract PVCs.

**Figure 1:**
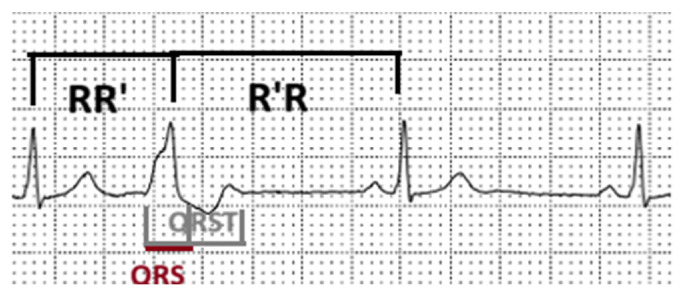
Measurements of coupling (RR’), postcoupling intervals (R’R), PVC QRS and QRST intervals (ECG samples from this study).

**Figure 2:**
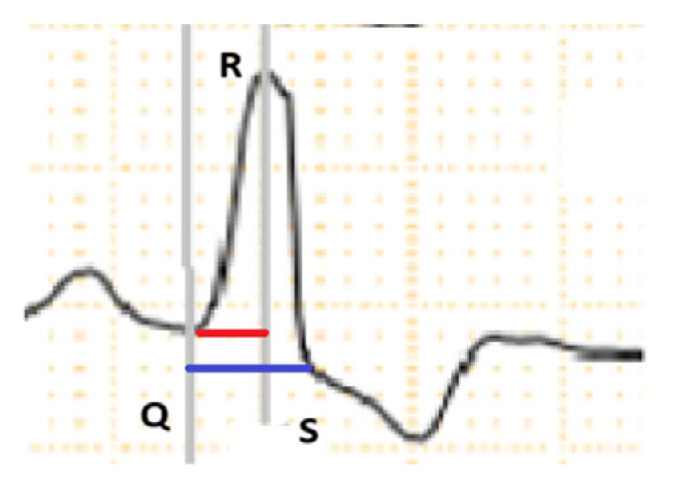
Measurements of maximum deflection index. Ratio between blue and red line (ECG samples from this study).

**Variant N3** (Table 1):

- Outflow tract PVCs:
  - LVOT (left ventricular outflow tract);
  - RVOT (right ventricular outflow tract);
- Left ventricular PVCs (papillary muscles, mitral valve, aortomitral region);
- Right ventricular PVCs (tricuspid valve, papillary muscles, moderator muscle);
- Fascicular PVCs;
- Atypical PVCs (epicardial).

(2) The following PVC parameters were measured and calculated in the recorded 12-lead ECGs:

- *Coupling interval* (RR’)—the duration in seconds from the R wave of the normal QRS complex to the R wave of the extrasystolic QRS complex (Figure 1);
- *Postcoupling interval* (R’R)—the duration in seconds from the R wave of the extrasystolic QRS complex to the R wave of the normal QRS complex (Figure 1);
- *Extrasystole (PVC) duration*—the duration of the extrasystolic (PVC) QRS and QRST (Figure 1);
- *Normal complex QRS duration*;
- Ratio between the PVC QRS and normal complex QRS (*ratio PVC/normal QRS*);
- *Maximum deflection index*, MDI—measured in precordial leads as the ratio between the shortest time from the beginning of the QRS to the R wave and the total QRS duration in V5-V6 derivations (Figure 2);
- *Heart rate* on ECG;
- *Other signs* *of ECG pathology* (hypertrophy, other rhythm and conduction disorders, repolarization changes in the sinus rhythm PQRST).

### 2.3. Outcomes

Primary outcomes included: (1) all-cause mortality; (2) life-threatening arrhythmias, including sustained ventricular tachycardia or ventricular fibrillation; and (3) the documented development of ventricular dysfunction, myocardial fibrosis, channelopathy, or cardiomyopathy identified through cardiogenetic testing performed as part of the study.

Secondary outcomes were documented as progression in the count of PVCs.

### 2.4. Statistical Analysis

Statistical analyses were performed using R (v. 4.2.2.). The distribution of quantitative variables was assessed using the Shapiro–Francia test. Normally distributed variables are presented as mean ± standard deviation, whereas non-normally distributed variables are expressed as median, minimal, and maximal values, and interquartile range (IQR). Categorical variables are reported as counts and percentages.

Comparisons between two groups were performed using Student’s *t*-test for continuous variables or the chi-square test, as appropriate according to data distribution. Comparisons among multiple groups were conducted using one-way analysis of variance (ANOVA) or the Kruskal–Wallis test, depending on distribution. Categorical variables were compared using the chi-square test or Fisher’s exact test. Correlations between variables were assessed using Pearson’s correlation coefficient or Spearman’s rank correlation coefficient, as appropriate.

A *p*-value < 0.05 was considered statistically significant.

## 3. Results

### 3.1. Participants and Procedures

According to the inclusion and exclusion criteria, 80 patients were included in this study: 42 (52.5%) boys and 38 (47.5%) girls. The median age of the patients was 12 years (minimum 5, maximum 17 years).

Most PVCs were diagnosed incidentally during a routine check-up; this was the case for 65 patients. General weakness was a complaint of five patients, while five experienced palpitations, four experienced chest pain, and one experienced syncope.

Cardiac MRI was performed for 30 patients. Abnormal results were detected in eight patients: five had ventricular dilatation, two of them also had a reduced LVEF, and three patients had myocardium fibrosis.

Nineteen patients underwent cardiogenetic testing. The results for seven patients were defined as abnormal, but some of the mutations had uncertain clinical significance: three were related to potassium channel mutations (KSNJ2; two KCNH2), one to sodium channel (SCN5A) mutations, one to an RyR2 mutation (associated with catecholaminergic polymorphic ventricular tachycardia), and two to cardiomyopathies (titin and laminin gene mutations).

The baseline characteristics are in Table 2.

### 3.2. 12-Lead-ECG

#### 3.2.1. ECG-Estimated Anatomical Localization

##### Variant N1: LBBB vs. RBBB PVCs

We classified the PVCs according to their morphology into LBBB and RBBB types.

LBBB PVCs were detected in 50 (62.5%) patients, with RBBB PVCs in 30 (37.5%) patients. RBBB PVCs were found more often in boys (RBBB 70% vs. LBBB 43.3%, *p* = 0.03), but no difference was observed in terms of age (median age RBBB 12 years vs. LBBB 13 years, *p* = 0.28). The PVC coupling and postcoupling intervals, PVC QRS duration, MDI, and heart rates on ECG did not differ significantly between the LBBB and RBBB groups (*p* > 0.05).

LBBB PVCs manifested more frequently (LBBB 8.2% vs. RBBB 5%, *p* = 0.02) and were more often complex (LBBB 64% vs. RBBB 33.3%, *p* = 0.009). We did not find a significant difference between the LBBB and RBBB morphologies for other variables, such as the manifestation of symptoms (asymptomatic: RBBB 80% vs. LBBB 83.3%, *p* = 0.78), a positive family history (*p* = 0.24), LVEF (RBBB median LVEF 61.9% vs. LBBB LVEF median 60.9%), multiform PVCs (LBBB 12% vs. RBBB 16.6%, *p* = 0.66), other ECG abnormalities, changes in cardiac MRI (RBBB two patients vs. LBBB six patients, *p* = 0.69), or abnormal cardiogenetic testing results (RBBB two patients vs. LBBB five patients, *p* = 0.69).

##### Variant N2: Outflow Tracts vs. Non-Outflow Tracts PVCs

We classified PVCs as outflow tracts in 44 (55%) cases and non-outflow tracts in 36 (45%) cases. Thus, more than half of the patients had an outflow tract PVC morphology.

Complex PVCs were most frequent with the outflow tract localization (outflow 65.9% vs. non-outflow 36.1%; *p* = 0.01). However, with this morphological classification, we did not find associations in terms of gender (*p* = 0.08), age (*p* = 0.44), absent symptoms (*p* = 0.2), a positive family history, calculated 12-lead ECG parameters (*p* > 0.05), LVEF (*p* = 0.3), or abnormalities on cardiac MRI (*p* = 0.2) or cardiogenetic tests (*p* = 0.23).

The initial (median 11.3% vs. 5.1%, *p* = 0.01) and maximal (12% vs. 6.8%, *p* = 0.01) PVC amounts per 24 h were higher for outflow tract PVCs than for non-outflow tract PVCs. Both PVCs morphologies tended to decrease.

##### Variant N3: Precisely Localized PVCs

The most probable localization of PVCs was in the outflow tracts, observed in 44 patients (55%). They were otherwise observed in the RVOT in 31 (38.8%), the LVOT in 13 (16.3%); the LV in 15 (18.8%), the RV in seven (8.8%), and the fascicules in 12 (15%) cases. In two patients (2.5%), the morphology of the PVCs was defined as atypical (potentially epicardial).

Fascicular PVCs were significantly more frequent in younger patients (*p* = 0.008). We did not find any significant associations for this ECG morphology according to probable localization with the results for other variables, such as gender (*p* = 0.052), absence of symptoms (*p* = 0.06), a positive family history (*p* = 0.07), LVEF (*p* = 0.46), multiform PVCs (*p* = 0.4), other ECG abnormalities, changes in cardiac MRI (*p* = 0.5), and abnormal cardiogenetic testing results (*p* = 0.38). Complex PVCs were most frequent in the outflow tract localization (65.9%, *p* = 0.04).

The widest PVC QRS complexes were observed in cases of atypical extrasystoles and RV-origin extrasystoles, while the narrowest were observed in cases of fascicular (median 80 ms) extrasystoles (*p* = 0.0002). An identical distribution was observed when the duration of the extrasystolic QRST complex was evaluated. The MDI estimate was significantly higher in cases of atypical extrasystoles (median 0.7; *p* = 0.0003).

According to PVC morphology, the largest number of PVCs occurred in the ECG-estimated RVOT localization; the median of the maximum PVC count per 24 h was 19.1% (*p* = 0.007), while the median of the initial PVC count per 24 h was 15.4% (*p* = 0.01). The PVC dynamics evaluated according to the ECG morphology showed that the daily number of PVCs of all morphological types tended to decrease.

#### 3.2.2. Other ECG Parameters

Complex PVCs were more common in patients with more frequent PVCs (non-complex vs. complex: initial PVC count, median 4% vs. 6.1% (*p* = 0.04); maximal PVC count, median 6.3% vs. 8.7% (*p* = 0.04)). However, patients with complex PVCs did not differ in terms of their age (*p* = 0.2), gender (*p* = 0.6), symptoms (*p* = 0.4), positive family history (*p* = 0.1), duration of extrasystoles (*p* = 0.06), coupling (*p* = 0.3) and postcoupling intervals (*p* = 0.07), MDI (*p* = 0.07), PVC/normal QRS ratio (*p* = 0.08), morphology, other ECG abnormalities (*p* = 0.9), LVEF (*p* = 0.5), genetic abnormalities (*p* = 0.49), or ventricular MRI abnormalities (*p* = 0.09).

We could not confirm that multiform PVCs were dependent on the patients’ age (*p* = 0.38), gender (*p* = 0.05), symptoms (*p* = 0.27), positive family history (*p* = 0.21), extrasystole duration (*p* = 0.4), coupling (*p* = 0.08) and postcoupling intervals (*p* = 0.34), MDI (*p* = 0.44), PVC/normal QRS ratio (*p* = 0.4), morphology, other ECG abnormalities (*p* = 0.8), genetic abnormalities (*p* = 0.65), or MRI abnormalities (ventricular dilatation. Dysfunction/myocardial fibrosis (*p* = 0.77).

We also could not prove any correlation of initial and maximal PVC counts with the coupling or postcoupling interval, MDI, PVC/normal QRS ratio, or LVEF (*p* > 0.05).

### 3.3. Outcomes

During our study, two patients experienced non-sustained tachycardia episodes related to physical activity. One patient was a 16-year-old boy without symptoms, with LV-morphology PVCs, fibrotic changes on MRI, normal genetics, and a maximal PVC count of 28.3%. The other was a 10-year-old girl with syncope episodes during physical activity, with RVOT- and RV-morphology multiform PVCs, a maximal PVC count of 15%, normal MRI results, and an RyR2 mutation (associated with catecholaminergic polymorphic ventricular tachycardia).

None of the patients experienced cardiac arrest during the study period.

We found that MRI abnormalities (ventricular dysfunction/dilatation/myocardial fibrosis) were associated only with the maximal PVC count—a median of 29.6% PVCs per 24 h (*p* = 0.04).

Furthermore, wider PVCs (QRS duration > 150 ms) were associated with MRI abnormalities (*p* = 0.008). We did not find an association between MRI abnormalities and multiform (*p* = 1) or complex (*p* = 0.26) PVCs, or a positive family history (*p* = 0.15). We also did not find a significant difference in ECG parameters, such as the heart rate, coupling and postcoupling intervals, MDI, or PVCs/normal QRS ratio (*p* > 0.05), with MRI abnormalities. MRI changes did not depend on the PVC morphology.

We did not find any reliable association regarding changes in cardiogenetic testing results and the patients’ age, gender, symptoms, PVC morphology, PVC counts, ECG parameters examined in this study, or cardiac MRI abnormalities (*p* > 0.05).

The prognosis regarding PVC count progression was not related to the patients’ age (*p* = 0.67), gender (*p* = 0.8), symptoms (*p* = 0.39), family history (*p* = 0.77), maximal (*p* = 0.3) or initial PVC (*p* = 0.73) count, heart rate (*p* = 0.65), coupling interval (*p* = 0.21), MDI (*p* = 0.27), PVC/normal QRS ratio (0.46), PVC morphological variant, or abnormalities found via MRI (*p* = 0.37) or genetic testing (*p* = 0.1). However, we detected that a longer postcoupling interval was strongly associated with PVC progression, with a cut-off value of 0.9 s (*p* = 0.002).

## 4. Discussion

This study was an attempt to determine the significance of ECG-estimated PVC morphological characteristics in terms of their clinical prognosis and outcomes in children and adolescents. It is well known that the morphology of QRS complexes on 12-lead ECGs can be utilized to determine the origin of PVCs [5].

LBBB-morphology PVCs are most often arisen in outflow tracts or in the right ventricle, while RBBB-morphology PVCs are likely to be lower in the left ventricle, fascicles, or epicardium (the latter localization potentially being prognostically worse). Cetin et al. studied the cases of 73 children with structurally healthy hearts and idiopathic PVCs. Among the groups of patients with LBBB and RBBB morphologies, monomorphic and multiform PVCs, dominant during the day and night, no reliable differences were found [23]. We found that LBBB-morphology PVCs were more often complex and associated with higher PVC counts. We did not find any significant association of this morphology with age, gender, symptoms, family history, or abnormalities on MRI or genetic tests.

It has been observed that PVCs with a RBBB morphology, or dominant S waves in leads II and III, are more often associated with fibrotic changes in the left ventricle on cardiac magnetic resonance imaging than ventricular extrasystoles with other morphologies [24]. There were only three confirmed cases of myocardial fibrosis in our study, so we cannot draw reliable conclusions. One of these patients had ECG-estimated RVOT PVC morphology (LBBB), one had ECG-estimated LV morphology (RBBB), and one had LBBB with a superior axis and confirmed mutation of the titin gene.

Outflow tracts are the most common sites of PVC foci accounting for more than two-thirds of all PVCs [5,25]. More than 50% of the PVCs in our study were ECG-estimated outflow tract PVCs. Outflow tract ventricular arrhythmias typically present in young patients and have notably higher PVC amounts [5]. In our study, the youngest patients had ECG-estimated fascicular PVCs. This morphological form of PVCs had the narrowest QRS complexes. According to data from Koester et al., an outflow PVC is classically a benign arrhythmia, but patients can be highly symptomatic [5]. In our study, we did not find a reliable relationship between clinical symptoms and the different morphological ECG-estimated variants (LBBB vs. RBBB; outflow vs. non-outflow) of extrasystoles. We also could not confirm a relationship between the coupling interval and the various ECG-estimated morphological classifications of PVCs.

Right ventricular outflow tract extrasystoles in adults are more often associated with malignant arrhythmias [26]. Seventy-five percent of PVCs that provoke ventricular fibrillation are found in the RV, RVOT, papillary muscles, or moderator muscle [27]. Two patients from our study demonstrated non-sustained ventricular tachycardia episodes with ECG-estimated LV and RVOT/RV multiform PVC morphologies. It has also been stated that threatening extrasystoles are characterized by a short coupling interval [27]. The patients in our study did not have a short coupling interval in single PVCs; this sign was only observed during non-sustained tachycardia episodes. None of the patients experienced cardiac arrest during the follow-up period.

It is thought that multiform and complex PVCs may be associated with primary cardiomyopathies or channelopathies [8]. We were unable to demonstrate any association of complex PVCs with abnormal results from MRI or genetic testing or with PVC count progression. However, cardiac MRI was performed according to indications in only 30 of 80 patients, while genetic testing was performed in 19 patients.

According to a recent systematic analysis from Flore et al., the occurrence of PVC-induced cardiomyopathy is associated with a high PVC burden [mean burden of 32.5% with 95% confidence interval (CI) 20.69–44.38% in pediatric patients with LV dysfunction vs. 15.47% with 95% CI 11.5–19.4% in patients without LV dysfunction] [4]. Pediatric studies present conflicting amounts of PVCs causing cardiomyopathy, ranging between 1.2 and 36% [7,28]. A recent retrospective study of Lithuania’s pediatric population revealed that the initial PVC counts on 24 h ECGs were not significant in that matter [28]. Ventricular dysfunction occurred in those with a median maximal PVC count per 24 h of 26.5% [28]. In this prospective study, we found that MRI abnormalities (ventricular dysfunction/dilatation/ myocardial fibrosis) were associated with the maximal PVC count only with a median of 29.6% PVCs per 24 h. In their systematic analysis, Flore et al. also reported that PVC-induced cardiomyopathy is associated with an older age and the male sex, while the predictive role of a short coupling interval, a longer QRS duration, and the presence of repetitive ventricular arrhythmias has not been consistently demonstrated across studies [4]. In our study, MRI abnormalities were also independent of the coupling or postcoupling interval, ECG-estimated PVC localization, age, and gender. In addition, we could not find a significant association between the ECG-estimated PVC morphological type and cardiogenetic testing results.

In adults, wide (QRS > 150 ms) PVCs are associated with a faster onset of left ventricular dysfunction; ventricular extrasystoles that are narrower, located closer to the Purkinje fibers, septal, and arise near the left bundle branch rarely cause left ventricular dysfunction [27,29]. Our study confirmed this theory: PVCs wider than 150 ms were strongly associated with all investigated MRI abnormalities. However, the systematic review by Flore et al. did not prove this theory [4]. On the other hand, in our study, the LVEF calculated via the Simpson method did not correlate with the PVC QRS duration. Nevertheless, it can be concluded that the PVC duration is more significant for the prognosis than the PVC ECG-estimated morphology itself. However, the findings should be interpreted cautiously given the relatively small cohort and limited number of adverse outcomes.

A recent study by Chueva et al. reported that idiopathic ventricular arrhythmias in children are frequently benign and may resolve spontaneously [3]. However, identifying which patients are likely to experience spontaneous resolution remains difficult, creating uncertainty regarding management strategies and the optimal timing of intervention [3]. Multivariable analysis identified four independent predictors of resolution: age younger than 12 years, isolated premature ventricular contractions without ventricular tachycardia, right-sided ectopic origin, and a 24 h PVC burden below 20% [3]. In our study, PVCs progression was only associated with the PVC postcoupling interval: a longer postcoupling interval was associated with a progressing PVC count. However, the postcoupling interval did not differ with gender, age, complex or multiform PVCs, MRI abnormalities, or genetic testing abnormalities.

From these results, we can state that in our population, it is appropriate to prescribe more detailed cardiac imaging studies when PVCs are wider than 150 ms and PVCs are more frequent than 26% per 24 h.

We assessed the strengths and limitations of this prospective observational study. We determined many parameters of PVCs: the localization of the focus using algorithms, evaluated the ECG-estimated PVCs morphology, and calculated specific ECG parameters, including the QRS duration of the extrasystoles, duration of the normal complex, ratio between the PVC and normal QRS complexes, maximum deflection time, coupling and postcoupling intervals, and other electrocardiographic markers of abnormalities.

A key limitation of our study is the potential for selection bias. Due to the limited number of patients who underwent cardiac MRI and cardiogenetic testing, only a small number of cases with ventricular dysfunction or myocardial fibrosis were identified, preventing proper assessment of these risk factors. In addition, because of the low number of abnormal MRI and cardiogenetic findings, multivariable modeling may not have been feasible.

Further research should focus on improving our understanding of the relationships between more detailed ECG parameters and computer modeling in population-based studies. However, we believe that assessing the morphology of extrasystoles may be clinically useful.

## 5. Conclusions

In this exploratory pediatric cohort, wider PVC QRS duration and higher maximal PVC burden were associated with ventricular MRI abnormalities, while longer postcoupling interval was associated with PVC progression.

## Figures and Tables

**Table 1:** ECG-estimated PVCs by the most probable localization according to the algorithms, ECG samples from this study [18,19,20,21,22]. RVOT—right ventricular outflow tract, LVOT—left ventricular outflow tract, RV—right ventricle, LV—left ventricle.

	Outflow Tracts		Non-Outflow Tracts			
	RVOT	LVOT	RV	LV	Fascicular	Epicardial
I	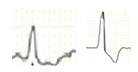	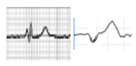	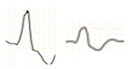	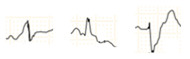	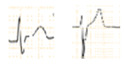	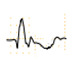
II	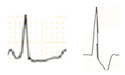	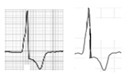	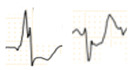	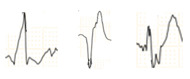	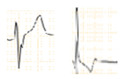	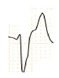
III	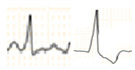	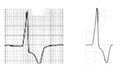	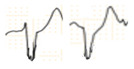	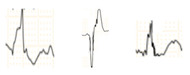	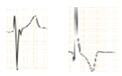	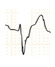
aVR	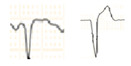	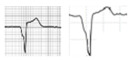	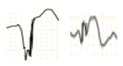	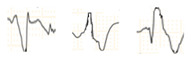	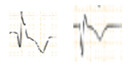	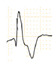
aVL	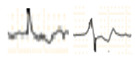	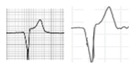	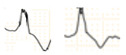	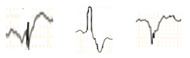	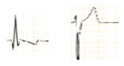	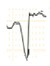
aVF	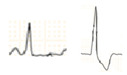	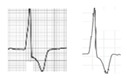	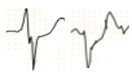	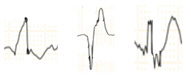	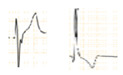	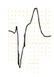
V1	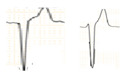	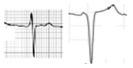	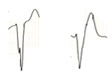	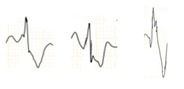	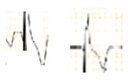	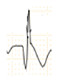
V2	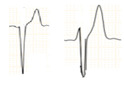	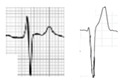	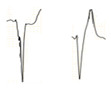	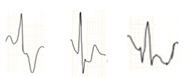	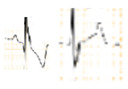	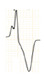
V3	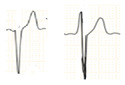	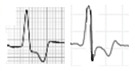	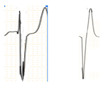	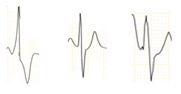	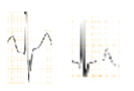	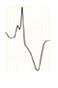
V4	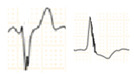	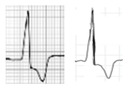	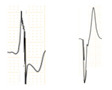	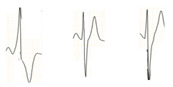	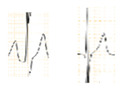	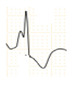
V5	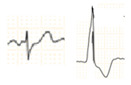	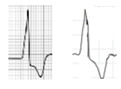	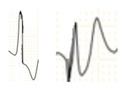	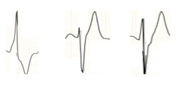	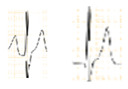	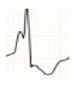
V6	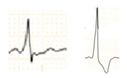	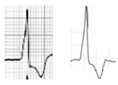	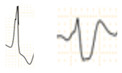	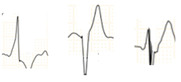	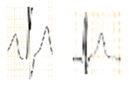	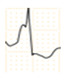

**Table 2:** Baseline characteristics of the patients.

Characteristics	
Boys, count (%)	42 (52.5%)
Age, years (IQR)	12 (10–15)
Asymptomatic, count (%)	65 (81.3%)
Positive family history, count (%)	21 (26.3%)
**12-lead ECG**	
Heart rate in ECG, beats/min, median (IQR)	110 (94–124)
Coupling interval, s (IQR)	0.4 (0.36–0.48)
Postcoupling interval, s (IQR)	0.64 (0.52–0.8)
Normal QRS duration, s, median (IQR)	0.06 (0.055–0.07)
PVC QRS duration, s, median (IQR)	0.12 (0.1–0.12)
PVC QRST duration, s, median (IQR)	0.32 (0.28–0.33)
PVC QRS/ normal QRS ratio (IQR)	2 (1.55–2.65)
MDI, median (IQR)	0.5 (0.25–0.5)
ECG signs of hypertrophy, count (%)	5 (6.3%)
ECG prolonged QTc interval, count (%)	5 (6.3%)
**Other**	
Follow-up time, months, median (IQR)	29 (14.8–46)
Initial PVCs count, %, median (IQR)	6 (3–16)
Maximal PVCs count, %, median (IQR)	9.2 (5.3–19.7)
Last 24 h ECG PVCs count, %, median (IQR)	5 (1.1–8.2)
Multiform PVCs, count (%)	11 (13.9%)
Complex PVCs, count (%)	42 (53.2%)
LV EF, %, median (IQR)	64.1 (60.6–68.1)
MRI abnormalities, count (%)	8/30 (26.7%)
Genetic abnormalities, count (%)	7/19 (36.8%)

## Data Availability

On request, the corresponding author will provide the datasets produced and/or analyzed during the current study.

## Abbreviations

The following abbreviations are used in this manuscript:

ACC	American College of Cardiology
AAP	American Academy of Pediatrics
AHA	American Heart Association
ASE	American Society of Echocardiography
HRS	Heart Rhythm Society
SCAI	Society for Cardiovascular Angiography and Interventions
SCCT	Society of Cardiovascular Computed Tomography
SCMR	Society for Cardiovascular Magnetic Resonance
SOPE	Society of Pediatric Echocardiography
ANOVA	One-way analysis of variance
ECG	Electrocardiogram
IQR	Interquartile range
LBBB	Left bundle branch block
LV	Left ventricle/left ventricular
LVEF	Left ventricular ejection fraction
LVOT	Left ventricular outflow tract
MDI	Maximum deflection index
MRI	Magnetic resonance imaging
PVC	Premature ventricular contraction
PVCs	Premature ventricular contractions
RBBB	Right bundle branch block
RV	Right ventricle/right ventricular
RVOT	Right ventricular outflow tract
RyR2	Ryanodine receptor type 2
